# Association of Hemodynamic Parameters with Clinical Outcomes in Cardiogenic Shock: Insights from Full-Flow Micro-Axial Flow Pump Data in a Retrospective Single-Center Study

**DOI:** 10.3390/jcm15083071

**Published:** 2026-04-17

**Authors:** Julia Riebandt, Roxana Moayedifar, Lukas Ruoff, Hebe Al Asadi, Sanja Söllner, Rabab Saleh, Oliver Seibert, Barbara Karner, Anne-Kristin Schaefer, Daniel Zimpfer, Thomas Schlöglhofer

**Affiliations:** 1 Department of Cardiac and Thoracic Aortic Surgery, Medical University of Vienna, 1090 Vienna, Austria; julia.riebandt@meduniwien.ac.at (J.R.); roxana.moayedifar@meduniwien.ac.at (R.M.); rabab.saleh@meduniwien.ac.at (R.S.);; 2 Center for Medical Physics and Biomedical Engineering, Medical University of Vienna, 1090 Vienna, Austria

**Keywords:** micro-axial flow pump, cardiogenic shock, Impella 5.5, hemodynamics, prediction

## Abstract

**Objectives:** The Impella 5.5 (J&J MedTech, USA) is increasingly used for refractory cardiogenic shock (CS), yet early predictors of mortality and recovery remain unclear. This study aimed to evaluate early patient characteristics and device-related parameters in relation to clinical outcomes; to compare outcome-based phenotypic groups (native heart recovery (NHR), heart replacement therapy (HRT), and death on the device (DEC)); and to analyze P-level impact on hemolysis and acute kidney injury. **Methods:** This retrospective single-center study included 28 CS patients supported with Impella 5.5 between May 2023 and August 2024. Data included intensive care unit (ICU) hemodynamics, vasoactive-inotropic score (VIS), lab markers, and pump parameters. Primary analysis evaluated early (first 24 h) parameters as potential indicators associated with mortality on the device and recovery, while secondary analyses compared hemodynamic and pump performance parameters across outcome groups, evaluated the association between P-level and hemolysis, and assessed the impact of shock etiology on clinical outcomes. **Results:** Among 28 patients (mean age 56 years, 10.7% female, body mass index (BMI) 27.7 kg/m^2^), NHR occurred in 39.3% and bridged to HRT in 42.9%. Non-survivors (17.8%) had significantly higher lactate (3.1 vs. NHR: 1.9 vs. HRT: 1.4 mmol/L, *p* < 0.001) and VIS (307.0 vs. NHR: 18.8 vs. HRT: 12.6, *p* < 0.001) at implantation. Higher VIS values (>69) were strongly associated with mortality on the device, with 100% sensitivity and 77% specificity (area under the curve (AUC) = 0.86); VIS < 9.9 was related to NHR (AUC = 0.63, 94% sensitivity, 45% specificity). P-levels were not linked to hemolysis index (r = −0.03, *p* = 0.64) or lactate dehydrogenase (r = −0.06, *p* = 0.37). **Conclusions:** Early vasoactive burden was associated with clinical outcomes in Impella 5.5-supported patients. No association between P-levels and the analyzed hemolysis surrogates was detected in this cohort. Distinct phenotypes across recovery outcomes may guide personalized management, but prospective validation of this exploratory and hypothesis-generating analysis is needed.

## 1. Introduction

The Impella 5.5 (J&J MedTech, USA) is a surgically implanted, micro-axial flow pump that provides full circulatory support and concomitant left ventricular unloading and has become an important treatment option for patients with refractory cardiogenic shock (CS) [1]. Despite the development and refinement of temporary mechanical circulatory support (MCS) over the past decade leading to its increasing adoption, early mortality in CS remains high (up to 50%), and clinical decision-making is hampered by limited evidence on early predictors of survival and recovery [2].

In theory, the Impella offers a superior hemodynamic support mechanism compared with other temporary MCS devices. However, non-randomized studies have found a much higher burden of adverse events for the Impella, and the one randomized trial also showed that the benefit of higher survival comes at the cost of more adverse events [3]. Importantly, the most recent investigations included earlier-generation devices, mixed cohorts combining Impella 5.5 with its precursor models, or pooled data from both percutaneous and surgically implanted devices. These methodological inconsistencies may have introduced bias and obscured device-specific outcomes, particularly given that the Impella 5.5 incorporates significant design modifications with potential clinical relevance. Identifying reliable prognostic markers is critical to refine patient selection, optimize timing of implantation, and individualize exit strategies such as native heart recovery (NHR), transition to durable heart replacement therapy (HRT), or palliative withdrawal.

Previous studies in mechanically supported CS populations have linked elevated lactate levels, impaired renal function, and high vasoactive-inotropic requirements as markers of poor outcome, while early hemodynamic improvement and effective ventricular unloading have been associated with better survival and NHR [4,5].

Recently, hemolysis has been a focus of interest regarding outcomes of further therapies such as durable left ventricular assist devices; however, pump data—such as continuous flow signals or performance level (“P-level”)—are rarely incorporated in clinical analysis, even though they may provide valuable mechanistic insights into unloading efficiency, device–patient interaction, and complication development [4].

Against this background, we designed a retrospective single-center analysis of Impella 5.5-supported patients to identify early patient-related predictors of mortality and NHR.

## 2. Materials and Methods

In this retrospective single-center study, all consecutive Impella 5.5-supported CS patients implanted between May 2023 and August 2024 were included. Exclusion criteria were the transfer of patients after implantation and missing datasets. A total number of 30 patient datasets were screened, and 28 went into the analysis while two were excluded for meeting the exclusion criteria. The study protocol was approved by the Institutional Review Board (EC Number: 1982/2024) with a waiver for informed consent.

For statistical analysis, Impella data (flow, P-levels), baseline demographics, vasoactive-inotropic score (VIS), and serial laboratory markers (lactate, hemolysis index, lactate dehydrogenase (LDH)) were collected and stored for postprocessing with MATLAB (R2024b, The MathWorks Inc., Natick, MA, USA), SPSS version 29 (IBM Corp., Armonk, NY, USA) and Stata BE version 19 (StataCorp LLC, College Station, TX, USA). Given the small sample size and exploratory, hypothesis-generating design, no formal sample size calculation was performed.

The primary aim of this study was to evaluate early (first 24 h) ICU and device-related parameters as potential indicators associated with subsequent clinical trajectory, including mortality on the device and NHR. The vasoactive-inotropic score (VIS), originally described by Gaies et al. as an extension of the inotrope score, has been widely adopted as a marker of hemodynamic support intensity and was calculated as: dopamine dose (μg/kg/min) + dobutamine dose (μg/kg/min) + 100 × epinephrine dose (μg/kg/min) + 10 × milrinone dose (μg/kg/min) + 10,000 × vasopressin dose (unit/kg/min) + 100 × norepinephrine dose (μg/kg/min) [6].

As secondary outcome analyses, patients were categorized into clinical trajectory groups: (i) death on Impella 5.5 support (deceased; DEC), (ii) NHR, and (iii) survival without recovery requiring transition to durable HRT. Across these groups, phenotypic differences in hemodynamics and pump parameters during the first and last 24 h of Impella 5.5 therapy, as well as the impact of P-level on hemolysis, were assessed. Furthermore, potential differences in CS etiology (acute myocardial infarction (AMI) vs. non-AMI) regarding outcomes and six-month survival were analyzed.

Categorical variables are expressed as numbers and percentages, continuous variables are expressed as mean ± standard deviation or median and interquartile range (IQR) as appropriate. The unpaired *t*-test or nonparametric Mann–Whitney U test were used for comparisons of continuous variables, while χ^2^ test or Fisher’s exact test were used for categorical variables, as appropriate. Predictive modeling was performed with ROC analysis (area under the curve, sensitivity, specificity, and maximal Youden index reported), and subgroup analyses conducted using correlation analysis and one-way ANOVA/Kruskal–Wallis tests, as appropriate. A *p*-value of < 0.05 was considered as significant.

## 3. Results

### 3.1. Patient Population

The mean patient age was 56 ± 14 years, and the proportion of female patients was 10.7% (n = 3). The primary etiologies of CS were acutely decompensated heart failure (ADHF) in 12 patients (42.9%) and acute myocardial infarction (AMI) in 11 patients (39.3%). The majority of the patients presented in SCAI stage E (60.7%) upon admission to our center.

Combined veno-arterial extracorporeal life support (ECLS) and Impella (“ECMELLA”) was utilized in 53.6% of patients (n = 15). Among these, 10 were on ECLS before Impella insertion. One patient was transitioned from a percutaneous Impella CP, whereas the remaining patients underwent primary Impella 5.5 implantation. The mean duration of Impella 5.5 support was 13.2 ± 6.9 days.

Detailed patient characteristics can be obtained from Table 1.

### 3.2. Short-Term Outcomes

Of the 28 patients included, 23 (82.1%) were successfully weaned from Impella 5.5 support, either through NHR (n = 11, 39.3%) or transition to HRT (n = 12, 42.9%). Among the bridged patients, seven (58.3%) underwent implantation of a durable ventricular assist device (VAD). The overall six-month survival rate among patients who were weaned or bridged was 78.3% (72.7% NHR; 83.3% HRT). These outcomes are presented in Table 2.

### 3.3. Outcomes Based on Etiology

Patient- and procedure-related parameters, as well as the outcomes, were comparable between the two groups with AMI-CS and non-AMI etiologies (including ADHF, post-cardiotomy CS (PC-CS)).

Within the AMI group, all patients were male, whereas there were 17.6% female patients in the non-AMI group (*p* = 0.258). Regarding co-morbidities, only a significantly higher incidence of history of myocardial infarction in the AMI-CS group (81.8% vs. 29.4%; *p* = 0.007) was observed.

In the non-AMI group, a higher proportion of patients could be weaned (47.1% vs. 27.3%), whereas in the AMI group, a higher proportion received a HRT (54.4% vs. 35.3%); however, none of these observations were significant (*p* = 0.435 and 0.441).

Six-month survival did not show any significant differences (AMI 72.7% vs. non-AMI 48.8%, *p* = 0.689).

### 3.4. Potential Predictors of Mortality on the Device and NHR

**Figure 1:**
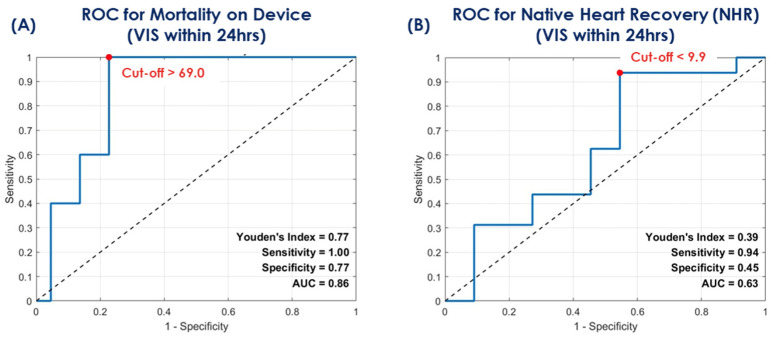
ROC analysis for mortality on the device (**A**) and native heart recovery (**B**). (VIS = vasoactive-inotropic score; ROC = receiver operator characteristics; AUC = area under the curve; dashed line = random guess).

VIS, as a known independent predictor of mortality in CS in pediatric and adult patient populations, was assessed within the first 24 h after implantation of the Impella 5.5. Predictive modeling using ROC analysis was performed and showed that a median VIS > 69.0 provided 100% sensitivity and 77% specificity [AUC = 0.86 (95% CI: 0.72–1.00)] as the optimal cut-off to differentiate the risk of meeting the primary endpoint of this study (mortality on the device). In contrast, a VIS < 9.9 [AUC = 0.63, 94% sensitivity, 45% specificity (95% CI: 0.39–0.86)] identified patients with a higher probability of achieving NHR within this cohort. Outcome stratified baseline data showed no significant differences between the three clinical trajectories. Details can be obtained from Table 3 and the results are depicted in Figure 1A,B.

### 3.5. Hemolysis and P-Levels in the Different Outcome Groups

Clinical and pump parameters were analyzed at the time of Impella 5.5 implantation and on the final day of support, and subsequently compared across the three outcome groups: NHR, HRT, and DEC.

Non-survivors demonstrated significantly higher median P-levels, both at implantation (8 [4] vs. NHR: 6 [4] vs. HRT: 6 [2]; *p* < 0.001) and at the end of support (7 [1] vs. NHR: 4 [1] vs. HRT: 6 [2]; *p* < 0.001).

**Figure 2:**
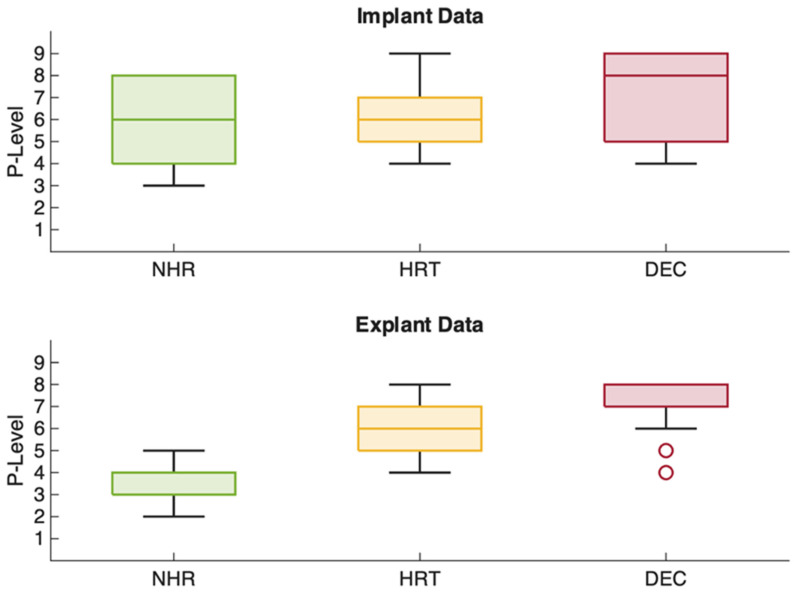
P-levels over the course of support. (P-level = performance level; NHR = native heart recovery; HRT = heart replacement therapy; DEC = deceased).

**Figure 3:**
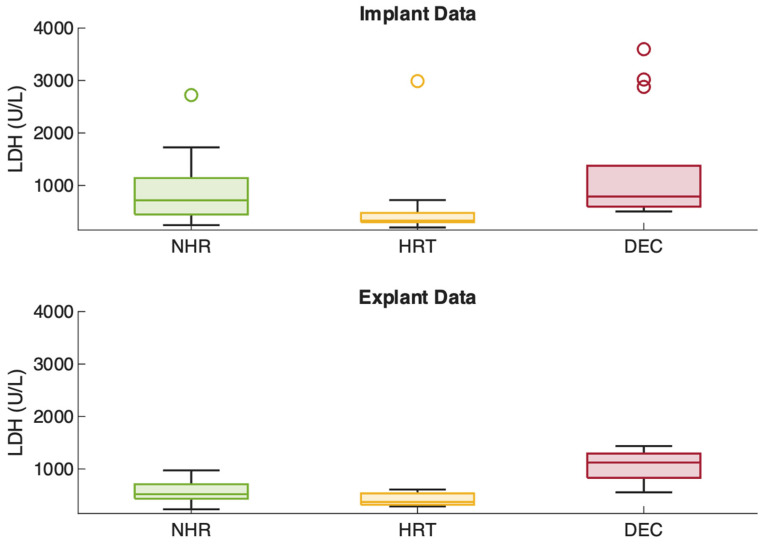
LDH over the course of support. (NHR = native heart recovery; HRT = heart replacement therapy; DEC = deceased).

**Figure 4:**
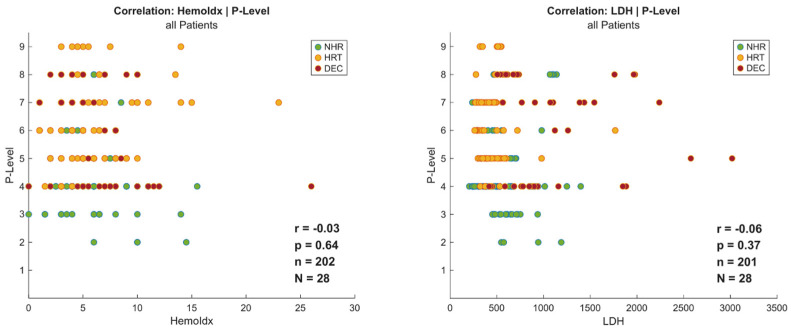
Scatter plot of hemolysis index (**left**) and LDH (**right**) in relation to P-level for all patients. (NHR = native heart recovery; HRT = heart replacement therapy; DEC = deceased; Hemoldx = hemolysis index; LDH = lactate dehydrogenase; N = number of evaluated patients; n = number of valid datapoints; p = statistical significance; r = correlation coefficient).

Similarly, LDH levels were significantly elevated in non-survivors at both time points (*p* < 0.001). DEC patients had the highest values at both implantation and explantation (843 to 968 U/L), nearly twice those of NHR (486.5 to 515.5 U/L) and HRT (lowest: 416 to 370 U/L). Additional details are presented in Figure 2 and Figure 3. Importantly, higher P-levels were not associated with an increase in hemolysis, represented by hemolysis index (r = −0.03, *p* = 0.64) and LDH (r = −0.06, *p* = 0.37) as institutional routine surrogate parameters for clinically relevant hemolysis (Figure 4).

Acute kidney injury requiring temporal renal replacement therapy occurred in five patients overall (17.6%), with three NHR patients and two deceased patients with mean P-levels of 6 at the time of implantation and 5 at the time of explantation.

Median pump flow on the day of implantation differed significantly among groups (*p* < 0.001), with values of 2.9 (2.3) L/min for NHR, 3.7 (1.0) L/min for HRT, and 4.0 (2.2) L/min for DEC. Over time, pump flow declined in the NHR and HRT groups but increased in DEC. The NHR group exhibited the largest median reduction to 2.2 (0.6) L/min, whereas HRT showed the numerically widest variability on the final day of support, with a median flow of 3.6 (2.3) L/min.

Median lactate declined significantly from implantation to explantation (*p* < 0.001) across all cohorts. DEC had the highest initial values (3.1 to 2.1 mmol/L), followed by NHR (1.9 to 1.2 mmol/L).

## 4. Discussion

In this retrospective single-center analysis of patients with refractory CS supported exclusively with the Impella 5.5, early patient and pump related markers were evaluated for whether they could predict mortality on device or NHR. We identified early vasoactive burden within the first 24 h as a discriminator of subsequent clinical trajectory. Most notably, the VIS demonstrated an association with mortality and NHR, suggesting that early hemodynamic stabilization under full-flow micro-axial support carries important prognostic information.

While prior studies in heterogeneous CS populations have linked elevated lactate levels, renal dysfunction, and vasopressor requirements to poor outcomes, most analyses combined different Impella generations or percutaneous and surgically implanted systems, limiting device-specific interpretation [4,5,7]. In a recent US registry analysis of Impella 5.5-treated patients (focusing on more than 14 days of support), the group found several pre-implantation parameters (renal markers, mean arterial pressure of 80 mmHg or more) to be linked to favorable outcomes (NHR or HRT). Also, the VIS assessed at pre-implantation and 72 h post-implantation correlated with mortality or the necessity of concomitant ECLS implantation if exceeding 12 (pre) or 63 (post) [8].

### 4.1. VIS as a Potential Early Decision-Enabling Marker

We observed that a VIS > 69 within the first 24 h was strongly associated with mortality on the device (AUC 0.86), whereas a VIS < 9.9 identified patients likely to achieve NHR. Persistently elevated vasoactive requirements despite Impella 5.5 support likely reflect ongoing circulatory instability and may identify patients with limited myocardial recovery potential. In this context, early VIS assessment may provide clinically useful information for early risk stratification beyond a simple risk description.

These findings may support a dynamic, parameter-guided treatment strategy. Patients with persistently high VIS despite full-flow support should undergo early reassessment of therapeutic goals, including the consideration of escalation strategies such as additional MCS (e.g., right ventricular support or combined ECLS approaches) and timely evaluation for permanent heart replacement therapies, such as durable VAD implantation, total artificial heart, or heart transplantation—according to institutional practice and local/national allocation systems.

Such an approach may help avoid a delayed transition to definitive therapy and reduce exposure to prolonged temporary MCS, which has been associated with increasing complication rates over time.

Another important observation derived from this dataset is the persistent tendency to initiate temporary MCS only after substantial clinical deterioration—the majority of patients were presented in SCAI stage E—underscoring the need for earlier recognition and intervention in the course of CS in order to enhance survival. This is in line with what the newest EACTS/STS/AATS guidelines are recommending—considering temporary MCS initiation when vasoactive requirements escalate (e.g., VIS > 20) in the absence of contraindications [9].

Our data may extend this paradigm, where the persistence of markedly elevated VIS despite Impella 5.5 support may identify a subgroup requiring early evaluation for HRT or escalation strategies, while a rapid reduction in vasoactive demand and support level may signal myocardial recovery potential. A structured, parameter-driven approach could help avoid both futile prolongation of temporary MCS and delayed transition to definitive therapy.

### 4.2. Device-Specific Insights: Integrating Pump Performance Parameters

A distinctive feature of this analysis is the integration of pump performance parameters into outcome assessment. In contrast to registry-based investigations, which rarely incorporate granular device metrics, we evaluated P-level and pump flow trajectories in relation to clinical endpoints.

Non-survivors exhibited higher P-levels and higher pump flows already at implantation, likely reflecting greater shock severity and the need for sustained maximal unloading. However, in this exploratory analysis, higher P-levels were not associated with increased hemolysis, as demonstrated by the absence of correlation with hemolysis index and LDH. The comparison of pump parameters and laboratory markers on the final day of support across outcomes must be interpreted with caution. These data points are fundamentally conditioned on the outcome itself and the highly variable duration of support. Consequently, these metrics function as end-state descriptors characterizing the terminal clinical phase rather than independent predictors of the clinical trajectory.

These observations may have clinical implications. While early-generation micro-axial devices such as the Impella CP or Impella 5.0 have been associated in some cohorts with hemolysis-related complications, including bleeding, transfusion requirements, and renal dysfunction [10,11], the Impella 5.5 features a modified design and full-flow characteristics. Our findings suggest that this platform may allow clinicians higher unloading strategies without proportionally increasing hemolytic risk, provided that appropriate positioning and right ventricular function are maintained [1,11,12]. Such an approach may contribute to improved outcomes, particularly in patients achieving NHR, as optimal unloading enhances the recovery process by reducing the myocardial wall tension and workload, leading to a reduced oxygen consumption and improved coronary perfusion [13]. In AMI-CS, it can reduce the ischemia–reperfusion injury and thereby potentially the infarct size [14].

### 4.3. Clinical Implications

In summary, the two principial clinical findings from this study suggest:

(1) VIS within the first 24 h of Impella 5.5 support shows a strong association with the clinical trajectory.
(2) Impella 5.5 P-level does not appear to be a primary driver of hemolysis, potentially suggesting that ventricular unloading may be titrated with a favorable safety profile regarding blood trauma.

In daily practice, VIS within the first 24 h after Impella 5.5 implantation may serve as a simple, readily available tool to:

− Identify patients with a high likelihood of NHR;
− Detect patients at a high mortality risk despite full unloading;
− Prompt timely evaluation for durable HRT.

Such an approach aligns with contemporary efforts to optimize the timing of advanced therapies in CS.

## 5. Limitations

This study is limited by its retrospective design, single-center setting, and relatively small sample size. Given the heterogeneity of the cohort and the lack of multivariable adjustment, residual confounding cannot be excluded. Therefore, the identified VIS thresholds should be interpreted as hypothesis-generating rather than definitive clinical cutoffs. Nonetheless, the strength of this analysis lies in the detailed integration of pump data with early clinical parameters in an exclusively Impella 5.5-treated cohort—data rarely available in large registries.

These observations highlight the need for larger prospective multicenter investigations to validate the current findings, and to better define the prognostic value of these early indicators in diverse clinical settings.

## 6. Conclusions

The Impella 5.5 represents an important temporary MCS option in CS, both as a stand-alone device and in combination with ECLS. In this cohort, 23 of 28 patients were successfully weaned from Impella 5.5 support or transitioned to durable HRT. Early vasoactive burden was associated with the clinical trajectory and may support early risk stratification in selected patients. No associations between high P-levels and the analyzed hemolysis surrogates were detected in this exploratory cohort. These are hypothesis-generating findings, and validation in bigger patient cohorts is needed.

## Figures and Tables

**Table 1:** Patient baseline demographics.

Baseline Demographics and Risk Factors	N (%) or Median (IQR)/Mean ± STD
BMI	27.7 (4.7)
Impella 5.5 Support (days)	13.2 ± 6.9
Age (years)	56.2 (14.4)
Gender	
Male	25 (89.3%)
Female	3 (10.7%)
Underlying Diseases	
Acute Myocardial Infarction (AMI)	11 (39.3%)
Acute Decompensated Heart Failure (ADHF)	12 (42.9%)
Other	5 (17.8%)
SCAI Stage	
D	7 (25.0%)
E	17 (60.7%)
Unknown	4 (14.3%)
Prior MCS	
ECLS	10 (35.7%)
Other Impella Model	1 (3.6%)
None	17 (60.7%)
Combination of Impella with ECLS (‘ECMELLA’ Therapy)	15 (53.6%)
Implantation Method	
Subclavian Artery/Axillary Artery	22 (78.6%)
Ascending Aorta	5 (17.9%)
Brachiocephalic Trunk	1 (3.6%)
NHR	11 (39.3%)
HRT	12 (42.9%)
VAD	7 (58.3%)
HTX	5 (41.7%)
DEC	5 (17.8%)
6-month survival (NHR and HRT)	18 (78.3%)

(BMI = body mass index; SCAI = Society for Cardiovascular Angiography & Interventions; MCS = mechanical circulatory support; ECLS = veno-arterial extracorporeal life support; NHR = native heart recovery; HRT = heart replacement therapy; VAD = ventricular assist device; HTX = heart transplantation; DEC = deceased).

**Table 2:** Summary of short-term outcomes.

Short-Term Outcomes	N (%)
NHR	11 (39.3%)
HRT	12 (42.9%)
VAD	7 (58.3%)
HTX	5 (41.7%)
DEC	5 (17.8%)
6-month survival (NHR and HRT)	18 (78.3%)

(NHR = native heart recovery; HRT = heart replacement therapy; VAD = ventricular assist device; HTX = heart transplantation; DEC = deceased).

**Table 3:** Outcome-stratified patient baseline demographics.

Demographics/Risk Factors	NHR	HRT	DEC	*p*-Value
Age	61 (24)	57.5 (18.5)	48 (27.5)	0.145
Sex				
Female	1 (9.1%)	1 (8.3%)	1 (20%)	0.778
Male	10 (90.9%)	11 (91.7%)	4 (80%)	0.778
BMI	24.7 (6.8)	27 (3.925)	30.9 (8.15)	0.055
Impella 5.5 Support Days	11.45 ± 4.987	15.67 ± 8.690	11 ± 4.919	0.279
Underlying Disease				
AMI	3 (27.3%)	6 (50%)	2 (40%)	0.611
Non-AMI	8 (72.7%)	6 (50%)	3 (60%)	0.611
SCAI Stage				
Stage D	4 (36.4%)	2 (16.7%)	1 (20%)	0.834
Stage E	6 (54.5%)	8 (66.6%)	3 (60%)	0.834
Unknown	1 (9.1%)	2 (16.7%)	1 (20%)	0.834
Prior MCS	4 (36.4%)	5 (41.7%)	2 (40%)	1.000
ECMELLA	4 (36.4%)	6 (50%)	5 (100%)	0.066

(BMI = body mass index; AMI = acute myocardial infarction; SCAI = Society for Cardiovascular Angiography & Interventions; MCS = mechanical circulatory support. Data are presented as N (%) or median (IQR)/mean ± STD).

## Data Availability

The data presented in this study are available on request from the corresponding author. The data are not publicly available due to privacy reasons; It’s patient data which cannot be posted public.
